# A Growing Concern: The Prevalence of Self-Medication in Pediatric Healthcare in India

**DOI:** 10.7759/cureus.53807

**Published:** 2024-02-07

**Authors:** Sohilkhan R Pathan, Vishal V Bhende, Kruti B Sharma, Vishal A Patel, Dinesh M Gangoda, Tanishq S Sharma

**Affiliations:** 1 Clinical Research Services (CRS), Bhanubhai and Madhuben Patel Cardiac Centre, Shree Krishna Hospital, Bhaikaka University, Karamsad, IND; 2 Pediatric Cardiac Surgery, Bhanubhai and Madhuben Patel Cardiac Centre, Shree Krishna Hospital, Bhaikaka University, Karamsad, IND; 3 Community Medicine, SAL Institute of Medical Sciences, Ahmedabad, IND

**Keywords:** parental care, children's health, risk factors, self-medication practices, pediatric healthcare

## Abstract

Self-medication, the practice of using medications without a valid prescription based on self-diagnosed symptoms, has become a global phenomenon, with a significant presence in developing nations like India. This inclination often arises from the desire to reduce healthcare costs and save time, though it carries inherent risks, including serious adverse effects and the potential masking of chronic disease symptoms. In India, the prevalence of self-medication varies widely, with factors such as media-driven advertisements, positive attitudes, and financial constraints contributing to its adoption, especially among lower- and middle-income families. The pediatric population in India is witnessing a notable increase in self-medication practices, driven by a mix of affordability, convenience, and limited awareness among parents. The risks associated with self-medication in pediatric healthcare are diverse, posing threats to developing immune systems and metabolisms in children. Antibiotic misuse further exacerbates concerns about antibiotic resistance, a global health crisis. Understanding the root causes of self-medication, including restricted healthcare access and societal pressures, is crucial for developing effective interventions. To address this issue comprehensively, a multifaceted approach is essential, emphasizing the need for widespread educational initiatives targeting healthcare literacy. Concurrently, reinforcing regulatory measures to monitor over-the-counter medication sales and conducting public awareness campaigns can deter unauthorized dispensing and promote responsible healthcare practices. Collaborative efforts involving healthcare providers, government bodies, pharmaceutical companies, and educational institutions are imperative to champion policies prioritizing children's health. It is a collective responsibility to ensure access to proper healthcare as an inherent right for every child in India. Urgent action is necessary to address the rising prevalence of self-medication, securing the well-being of the younger generation and paving the way for a healthier and more resilient future.

## Editorial

The practice of self-medication has a long-standing history on a global scale, exerting significant influence in developing nations such as India. As per the World Health Organization, self-medication encompasses addressing illnesses and providing initial aid in daily life. Consequently, in developing countries, pharmaceutical establishments frequently act as the initial point of contact for healthcare [1-2].

Self-medication is the practice of using drugs to address self-diagnosed disorders or symptoms or the occasional or consistent use of a prescribed medication for chronic or recurrent diseases or symptoms. This age-old practice has witnessed growth for various reasons, including the desire for self-care, a sense of empathy towards family members during illnesses, limited access to healthcare services, poverty, lack of awareness, misconceptions, extensive drug advertisements, and the availability of medications in establishments beyond pharmacies. Key sources of self-medication include relatives, friends, and pharmacists who not only provide medications but also information about their use. Simultaneously, factors such as limited access to healthcare, physician fees, time constraints, a lack of trust in physicians, and inadequate enforcement of drug laws have been identified as influencing factors in the behavior of self-medication [3-4].

In recent years, there has been a noticeable surge in the prevalence of self-medication practices among the pediatric population in India. In India, the prevalence of self-medication practices spans a wide range, from 8.3% to 92%. Notably, in countries like India, the primary drivers behind the escalating trend of self-medication are media and internet advertisements, despite violating the Drugs and Magic Remedies Act of 1954. Additionally, factors such as a positive attitude and confidence in both the drug and the understanding of the disease condition contribute significantly to the adoption of self-medication practices. A notable trend is that self-medication is more common among lower and middle-income families than among high-income families. This is attributed to the inability of individuals from lower-income levels to afford medical health insurance coverage plans, leading them to rely on self-medication as a practical alternative. Positive attitudes and confidence concerning both the drug and the perceived ailment are significant driving factors behind the adoption of self-medication. The inability of individuals from lower income brackets to afford medical health insurance coverage plans further accentuates the commonality of self-medication, particularly among lower and middle-income families compared to their higher-income counterparts [5]. While the ability to access over-the-counter medications provides convenience to parents and caregivers, it also raises serious concerns regarding the potential risks and adverse effects on children's health. This growing trend demands immediate attention and comprehensive strategies to address the underlying issues that contribute to this risky behavior.

In India, a country known for its rich tapestry of cultural traditions and varying levels of healthcare accessibility, there is a noticeable trend of parents turning to self-medication for their children. Whether dealing with common ailments like colds and fevers or facing more serious health issues, caregivers often choose over-the-counter medications without consulting healthcare professionals. This practice, driven by factors such as affordability, convenience, and lack of awareness, poses significant risks to the well-being of the younger generation. The dangers associated with self-medication in pediatric healthcare are numerous. Children, in their crucial formative years, have developing immune systems and metabolisms that respond differently to medications compared to adults. Incorrect drug dosages, misguided diagnoses, and the absence of professional medical guidance can lead to adverse reactions, complications, and long-term health issues. It is crucial to address this issue comprehensively to safeguard the health and safety of our children.

Additionally, the improper use of antibiotics in self-medication exacerbates the alarming rise in antibiotic resistance, a global health crisis that jeopardizes our ability to effectively combat common infections. The excessive and incorrect application of antibiotics in pediatric care poses a significant threat to the effectiveness of these medications, making children more vulnerable to infections that were once easily treatable. Understanding the root causes of the prevalence of self-medication is crucial for formulating effective interventions. Factors such as limited access to healthcare facilities, financial constraints, insufficient healthcare literacy, and societal pressure to quickly address illnesses collectively contribute to parents' inclination towards self-medication. Moreover, the lack of stringent regulations governing the sale of over-the-counter drugs worsens the situation. To address this issue at its core, a multifaceted approach is essential. Firstly, there is an immediate need for widespread educational initiatives aimed at enhancing healthcare literacy among parents and caregivers. These initiatives should emphasize the importance of consulting qualified healthcare professionals for precise diagnoses and the implementation of appropriate treatment plans.

**Figure 1:**
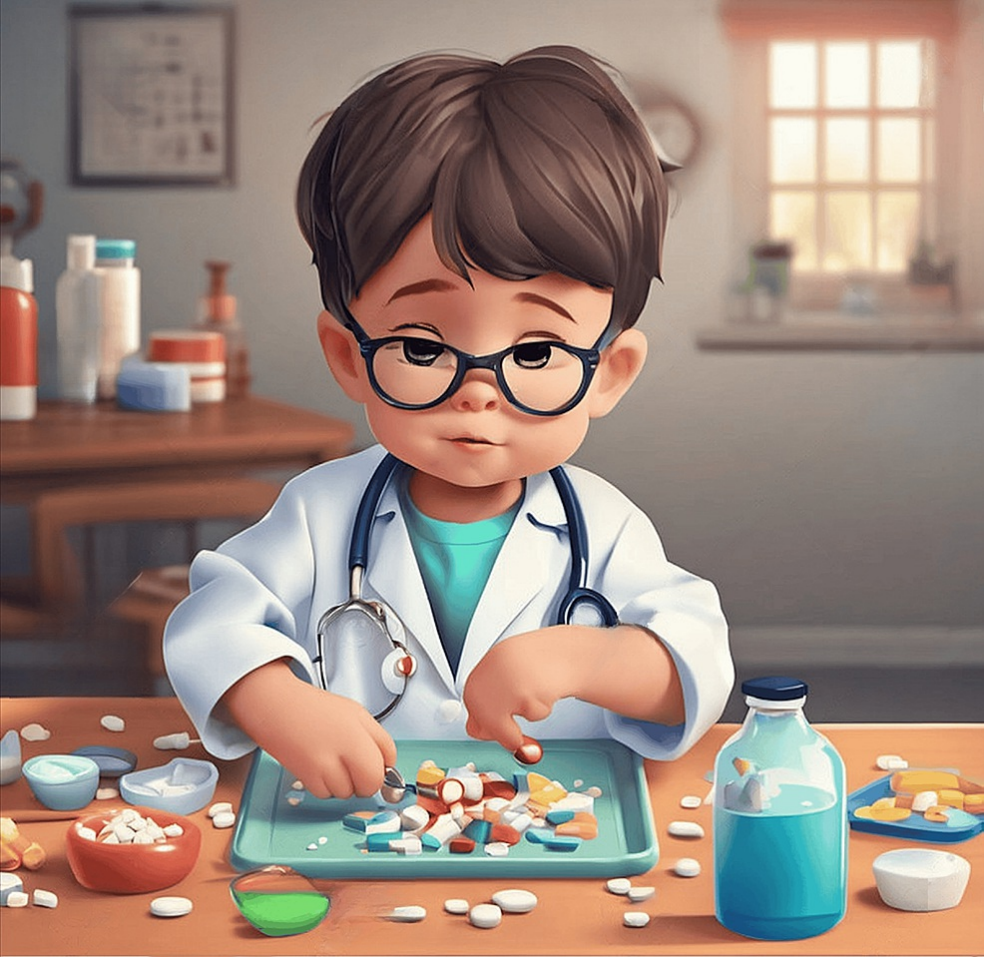
Imaginary example of child self-medication animation. The image was created by Sohilkhan R. Pathan on February 01, 2024, using OpenAI's AI-Generated Image Exploring [123RF AI Generator]. Retrieved from https://www.123rf.com/.

At the same time, there is an urgent need to strengthen regulatory measures to closely monitor and control the sale of over-the-counter medications. Reinforcing the enforcement of existing regulations, coupled with concerted public awareness campaigns, can serve as deterrents against the unauthorized dispensing of medications and promote a culture of responsible healthcare practices. Effectively tackling the prevalence of self-medication in pediatric healthcare requires collaborative efforts involving healthcare providers, government bodies, pharmaceutical companies, and educational institutions. A united front can advocate for policies that prioritize the health and well-being of children, asserting that access to proper healthcare is an inherent and nonnegotiable right for every child in India. Through collective action, we can lay the groundwork for a healthcare landscape that safeguards the welfare of our younger generation (Figure 1).

Conclusions

Addressing the rise of self-medication in pediatric healthcare in India requires a swift, multifaceted approach. Understanding the root causes, implementing targeted educational campaigns, and strengthening regulatory measures are key components. Thorough research is essential to comprehend the factors contributing to self-medication, including limited healthcare access and financial constraints. This insight forms the basis for effective interventions. Educational initiatives play a crucial role, emphasizing the risks of self-medication and promoting the importance of consulting healthcare professionals for accurate diagnoses and tailored treatment. Simultaneously, enforcing and enhancing regulations on over-the-counter medications can act as a deterrent. Coupled with public awareness campaigns, this can encourage responsible healthcare practices. Collaboration between healthcare providers, government bodies, pharmaceutical companies, and educational institutions is vital. Advocating for policies prioritizing children's health ensures access to proper healthcare as a nonnegotiable right. Embracing this collective responsibility paves the way for a future generation marked by robust health and resilience, shaping a healthier tomorrow for India's children.

## References

[1] Goyal A, Gaur A, Chhabra M (2018). Knowledge, attitude and practices of over the counter (OTC) medicines among rural population - a cross sectional study. Asian J Pharm Pharmacol.

[2] Panda A, Pradhan S, Mohapatro G, Kshatri JS (2017). Predictors of over-the-counter medication: a cross-sectional Indian study. Perspect Clin Res.

[3] Ahmad A, Patel I, Mohanta G, Balkrishnan R (2014). Evaluation of self-medication practices in rural area of town Sahaswan at northern India. Ann Med Health Sci Res.

[4] Limaye D, Limaye V, Krause G (2017). A systematic review of the literature to assess self-medication practices. Ann Med Health Sci Res.

[5] Rashid M, Chhabra M, Kashyap A, Undela K, Gudi SK (2020). Prevalence and predictors of self-medication practices in India: a systematic literature review and meta-analysis. Curr Clin Pharmacol.

